# The relationship between physical activity and mental toughness among Chinese university students: the chain-mediated role of self-esteem and social support

**DOI:** 10.3389/fpsyg.2025.1592192

**Published:** 2025-07-07

**Authors:** Zhixing Zhou, Jun Xiang

**Affiliations:** School of Physical Education and Health, Zhaoqing University, Zhaoqing, China

**Keywords:** physical activity, mental toughness, self-esteem, social support, university students

## Abstract

**Background:**

As Chinese university students increasingly face psychological challenges such as academic pressure and employment-related stress, mental toughness has gained attention as a crucial trait for coping with adversity. Physical activity has been considered a potential way to enhance mental toughness, yet its underlying mechanisms remain insufficiently understood.

**Objective:**

This study aimed to examine the associations between physical activity and mental toughness among university students, and to explore the potential mediating roles of self-esteem and social support.

**Methods:**

This study used the Physical Activity Scale, the Self-Esteem Scale, the Social Support Scale, and the Mental Toughness Scale with 1,184 university students in Guangdong Province as the research subjects. Data were analyzed sequentially using Pearson correlation analysis, structural equation modeling test and bias-corrected percentile bootstrap.

**Results:**

(1) Physical activity was positively correlated with mental toughness (*r* = 0.216), and the direct path between physical activity and mental toughness was significant (*β* = 0.085, *p* < 0.01, CI [0.04, 0.13]); (2) physical activity was positively correlated with self-esteem (*β* = 0.173, *p* < 0.01, CI [0.11, 0.23]) and social support (*β* = 0.063, *p* < 0.05, CI [0.08, 0.11]); self-esteem was positively correlated with social support (*β* = 0.397, *p* < 0.01, CI [0.34, 0.45]) and mental toughness (*β* = 0.350, *p* < 0.01, CI [0.30, 0.40]); and social support was positively correlated with mental toughness (*β* = 0.304, *p* < 0.01, CI [0.25, 0.35]); and (3) self-esteem and social support played a significant mediating role between physical activity and mental toughness. The mediating effect consisted of 3 pathways: physical activity → self-esteem → mental toughness (mediating effect value of 0.061), physical activity → social support → mental toughness (mediating effect value of 0.019), and physical activity → self-esteem → social support → mental toughness (mediating effect value of 0.021).

**Conclusion:**

The findings suggest that physical activity is positively associated with mental toughness among university students, and this relationship is partially mediated by self-esteem and social support. Given the cross-sectional nature of the study, conclusions about causality cannot be drawn.

## 1 Introduction

In the current social context, the college student population, due to their unique life environment and developmental stage, is faced with a variety of mental pressures and psychological challenges, including academic pressure, future employment concerns, interpersonal relationship management, and self-identity (Wu et al., 2020). As an individual’s ability to adapt effectively and flexibly in the face of adversity, setbacks, or major threats, mental toughness has become an important psychological resource for coping with these challenges (Li et al., 2024). Research has shown that individuals with high mental toughness are better at applying a variety of coping strategies to reduce stress, exhibit lower levels of anxiety and depressive symptoms, and reduce aggressive behaviors when confronted with stressful events (Jiang et al., 2023; Gloria and Steinhardt, 2016). However, the formation of mental toughness is influenced by a variety of factors such as genetics, environment, and personal experiences, of which physical activity is considered to be one of its key predictors. University students who regularly engage in physical activity are able to significantly reduce anxiety and depressive symptoms, and exhibit higher levels of mental toughness and better mental health (Li and Guo, 2023; Rodríguez-Romo et al., 2022). Nonetheless, research on how physical activity affects university students’ mental toughness through psychological mechanisms is still insufficient, and in particular, the mechanisms by which self-esteem and social support play a role have not been fully explored. Therefore, it is of great theoretical and practical significance to explore the relationship between physical activity and university students’ mental toughness and its mechanisms.

Self-esteem, as an individual’s positive evaluation of self-worth, may influence an individual’s mental toughness and help him or her cope with stress better (Auttama et al., 2021). Social support, on the other hand, further promotes an individual’s mental toughness by providing emotional, informational, and instrumental support (Southwick et al., 2016). Research has also shown that physical activity not only directly positively associated with an individual’s self-esteem level, but also indirectly associated with mental toughness through social support networks (Wei et al., 2024). In summary, on the basis of previous studies on university students’ mental toughness, this study, from the perspective of physical exercise, intends to explore its relationship with university students’ mental toughness and its mechanism of action, and focuses on the chain mediating role of self-esteem and social support, with a view to enriching the theoretical framework of mental toughness, providing empirical evidence for the mental health education in colleges and universities, and helping university students better cope with the various challenges of their lives.

## 2 Literature review and research hypotheses

### 2.1 Physical activity and mental toughness

Physical exercise, as an active and healthy lifestyle, has been widely recognized as having a significant positive impact on an individual’s physical and mental health and behavioral patterns. Research has pointed out that physical activity not only improves physical fitness but also has a positive effect on mental health through physical movement practice and exercise load, especially in the college student population (Herbert, 2022). First of all, studies have shown that there is a close positive correlation between physical activity and mental toughness. Mental toughness refers to an individual’s capacity to adapt effectively and flexibly when facing adversity, setbacks, or significant stressors. Physical activity has been widely recognized as a key factor associated with the development and enhancement of mental toughness (Zhou and Zhou, 2022). Emerging evidence suggests that engaging in physical activity may contribute to improved mental toughness by promoting positive mood states and alleviating symptoms of depression and anxiety (Husain et al., 2024). Both the frequency and intensity of physical activity appear to play important roles in shaping individuals’ psychological resilience. Specifically, engaging in moderate-intensity physical activity on a weekly basis has been associated with significant improvements in mental toughness among university students (White et al., 2017). Moreover, Stoltz (1997, 2000) Adversity Quotient (AQ) theory provides a robust framework for understanding how physical activity fosters mental toughness. Physical activity enhances perceived control by offering structured, goal-oriented challenges (e.g., completing a 5 K run or achieving a personal fitness milestone). These tasks require planning, effort, and self-regulation, which reinforce an individual’s belief in their ability to influence outcomes. For instance, studies suggest that regular exercisers are more likely to adopt problem-focused coping strategies under stress, aligning with high-AQ individuals’ tendency to prioritize actionable solutions over helplessness (Nimitniwat, 2011). Thus, physical activity appears to be related to mental toughness both directly and through its associations with emotional regulation. Therefore, Hypothesis 1 of this study is proposed: physical activity is positively associated with mental toughness.

### 2.2 The mediating role of self-esteem

Self-esteem, as an individual’s positive evaluation of self-worth, is one of the key indicators of positive psychology and there is a strong positive correlation between it and mental toughness. Self-esteem has been found to be positively associated with mental toughness traits such as self-acceptance, self-responsibility, and self-maintenance, which may contribute to individuals’ ability to cope more effectively with stress and challenges (Doğrusever et al., 2022). Previous research suggests that individuals with high self-esteem tend to exhibit greater mental toughness when facing adversity (Chen et al., 2022). Conversely, low self-esteem has been associated with reduced mental toughness in challenging situations (Basson, 2021), highlighting the potential role of self-esteem as a psychological resource in coping with stress. Therefore, self-esteem is not only an important component of mental toughness, but also plays a key role in constructing and maintaining mental toughness. Moreover, According to Self-Determination Theory (SDT), individuals have three basic psychological needs—autonomy, competence, and relatedness—which are essential for psychological growth, integrity, and well-being (Deci and Ryan, 2000). Within the SDT framework, self-esteem is considered a potential psychological pathway through which physical activity may be associated with mental toughness. Participation in physical activity is often accompanied by experiences of competence and mastery, which are positively related to self-esteem. In turn, individuals with higher self-esteem tend to report greater levels of mental resilience, suggesting a possible link between these constructs. Furthermore, previous studies have indicated that physical activity may be associated with greater mental toughness, possibly through its positive relationship with self-esteem (Kim and Ahn, 2021; Husain et al., 2024). Therefore, Hypothesis 2 of this study is proposed: self-esteem mediates the relationship between physical activity and mental toughness.

### 2.3 Mediating role of social support

One of the mediating mechanisms in this study is the mediating role of social support. Social support mediates the relationship between physical activity and mental toughness. First, studies have shown that social support, as an important resource acquired by individuals from social networks, has a significant impact on the enhancement of university students’ mental toughness because social support can effectively alleviate the negative emotions of individuals in the face of stress, thus enhancing their mental toughness (Wang and Bu, 2023; Lin and Xiong, 2020). The Buffer Hypothesis states that social support can play a buffering role in the face of stress and reduce the negative impact of stress on mental health. For university students, academic and life stresses are common stressors, and good social support can help them better cope with these stresses and thus maintain a high level of mental toughness (Rex, 2023). Second, physical exercise has been recognized not only as a key approach to improving physical fitness, but also as a potential avenue for gaining social support. A growing body of research suggests that university students who participate in team sports tend to report higher levels of perceived social support, which has been associated with improved athletic performance and psychological well-being (Van Luchene and Delens, 2021; Eather et al., 2023). Moreover, recent findings indicate that for university students, engaging in physical activity may support the development of mental toughness, potentially by facilitating access to social support that helps them better navigate academic and life-related stressors (Husain et al., 2024). Therefore, Hypothesis 3 of this study is proposed: social support mediates the relationship between physical activity and mental toughness.

### 2.4 Chain mediating role of self-esteem and social support

**Figure 1:**
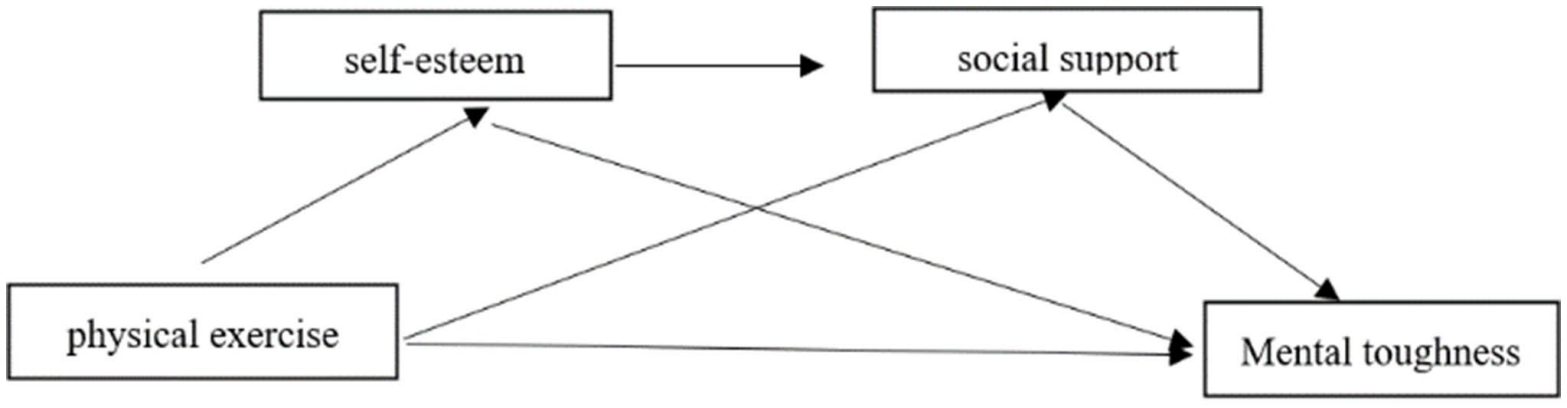
Research framework diagram.

Self-esteem and social support have been identified as potential sequential mediators in the relationship between physical activity and mental toughness among university students. Prior studies have reported a significant positive association between self-esteem and perceived social support, suggesting that the two may be mutually reinforcing and together contribute to individuals’ mental well-being. It has been proposed that higher levels of self-esteem—as a positive evaluation of one’s self-worth and competence—may be linked to greater confidence in social interactions, which in turn is associated with increased access to social support (Karunarathne, 2022). The Buffer Hypothesis model suggests that individuals with high self-esteem are more inclined to actively seek social support and better utilize these support resources in the face of stress, which ultimately improves their ability to cope with stress (Zhang et al., 2024). In addition, the study also pointed out that individuals who participate in physical activity tend to have higher levels of self-esteem, which in turn can help them obtain more social support, thus further enhancing Mental toughness (Li et al., 2023; Deng et al., 2022). Therefore, Hypothesis 4 is proposed: self-esteem and social support act as chain mediators between physical activity and mental toughness among university students. In summary, this study constructed a chain mediation model (see Figure 1) with physical activity as the independent variable, mental toughness as the dependent variable, and self-esteem and social support as the mediating variables, with a view to providing theoretical guidance for university students’ mental health interventions.

## 3 Research objects and methods

### 3.1 Research subjects

This study used stratified random sampling method to randomly select undergraduate university students of different grades in 10 universities in Guangdong Province in March 2024 as the research subjects, and a total of 1,280 questionnaires were distributed. Inclusion criteria included: enrolled undergraduate university students; aged between 18 and 23 years old; and physically healthy and without disabilities. The exclusion criteria included: not enrolled undergraduate students; age not meeting the requirements; the presence of physical disabilities or poor health. According to the inclusion and exclusion criteria, 1,184 valid questionnaires were finally recovered, with a validity rate of 92.5%. The mean age of the subjects was 20.09 ± 1.20 years, including 542 male and 642 female; 355 in the first year of university, 332 in the second year of university, 364 in the third year of university, and 133 in the fourth year of university. There was no significant difference between the variables of different genders and ages. The research process of this study strictly followed the ethical requirements of the Declaration of Helsinki, and the study was supported and approved by the Institutional Review Board of Zhaoqing University (2024016). All participants were informed of the purpose and characteristics of the study and signed an informed consent form, emphasizing the voluntary nature of participation and data confidentiality. The questionnaire survey was conducted on-site using the “Questionnaire Star” platform, and the testers were professionally trained university sport psychology students and faculty members to ensure the standardization of the survey process and the reliability of the data, as well as to control for age, gender, and other variables, so as to ensure the accuracy of the results of the study.

### 3.2 Research methods

#### 3.2.1 Questionnaire measurement method

##### 3.2.1.1 Physical exercise

This study used Liang (1994) Physical Activity Rating Scale (PARS-3) for measurement, which mainly consists of physical activity intensity (e.g., what is the intensity of your physical activity?), duration of each exercise session (e.g., How many minutes at a time do you perform physical activity at the above intensity?) and frequency of exercise (how many times a month do you engage in the above physical activities?) 3 dimensions were composed (Liang, 1994). The total score was calculated by the formula “Physical Activity = Intensity × (Exercise Time-1) × Exercise Frequency.” Each score was divided into 5 levels and scored from 1 to 5. The maximum score for physical activity is 100 points, and the minimum is 0 points. Physical activity was categorized as follows: small exercise ≤19 points, medium exercise 20–42 points, and large exercise ≥43 points. This is a well-established scale that has been widely used to assess physical activity among university students (Hou, 2024; Li et al., 2022). It has demonstrated acceptable reliability and validity in prior research and is considered appropriate for the population investigated in this study. The items are behaviorally specific and well-suited to capturing the typical patterns of physical activity among university students (Hou, 2024; Li et al., 2022). In this study, the total score of the physical activity level scale was used to represent the physical activity level of university students, and the Cronbach’*α* coefficient of the scale was 0.62, with reliable reliability.

##### 3.2.1.2 Self-esteem

Self-esteem was measured in this study using the Rosenberg Self-Esteem Scale (Rosenberg, 1965), which was translated by Ji and Yu (1993). The scale is currently the most widely used self-esteem measurement tool and is divided into “self-worth” (e.g., “I feel that I have many good qualities”), “self-acceptance” (e.g., “On the whole, I feel that I have many good qualities”), and “self-esteem” (e.g., “I feel that I have many good qualities”). “Overall, (I feel satisfied with myself”), with 10 questions. A 4-point scale (entries 3, 5, 8, 9, and 10 reverse scored) was used from 1 (very much not) to 4 (very much). It has been pointed out that item 8, “I wish I could earn more respect for myself,” has cultural differences between Chinese and Western cultures and should be scored positively or deleted, so this item was scored positively in this study. The total score of the scale ranges from 10 to 40, with higher scores indicating higher levels of overall self-esteem. The scale has been shown to have high applicability in the Chinese college student population (Hou, 2024). In this study, the Cronbach’s alpha coefficient of the scale was 0.94, which is reliable.

##### 3.2.1.3 Social support

This study measured social support using the Social Support Scale for university students developed by Ye and Dai (2008). The scale includes subjective support (e.g., most of my classmates are very supportive of me), objective support (e.g., I can rely on my family or relatives and friends in times of difficulty), and support utilization (e.g., I will take the initiative to ask for help from others when faced with difficulties.) 3 dimensions with 17 entries. The scale is scored on a 5-point Likert scale, with the 5 options being “not met, somewhat not met, not sure, somewhat met, and met,” with a higher total score indicating a higher level of overall social support and its dimensions. The scale has been shown to have good reliability and validity in the measurement of social support among Chinese university students (Ou et al., 2023). In this study, the Cronbach’s alpha coefficient of the scale was 0.95, indicating reliable reliability.

##### 3.2.1.4 Mental toughness

The CD-RISC questionnaire adapted by Campbell-Sills and Stein (2007) with 10 items (e.g., I can adapt when things change) was used in this study to assess the overall level of Mental toughness of individuals (Campbell-Sills and Stein, 2007). The scale has a one-factor structure and is scored on a 5-point Likert scale, i.e., 0 for “never,” 1 for “seldom,” 2 for “sometimes,” and 2 for “often.” “Often” scores 3 and ‘Always’ scores 4. The total score ranges from 0 to 40, and the higher the total score, the stronger the mental toughness of the individual. The scale has been shown to have good reliability and validity in the measurement of social support among Chinese university students (Zeng et al., 2023). In this study, the Cronbach’s alpha coefficient of the scale was 0.94, indicating reliable reliability.

#### 3.2.2 Mathematical and statistical methods

After the questionnaire data were recovered, all questionnaires were subjected to validated factor analysis as well as data common method bias test using SPSS26.0. IBM SPSS26.0 was used for descriptive statistics, and independent samples *t*-test was conducted for gender to understand the variability of different genders among the variables; one-way ANOVA was used to compare the variability of different ages, among the variables; and then Pearson correlation analysis was conducted to calculate the relationship among physical activity, self-esteem, social support, and mental toughness, and normally distributed continuous variables were expressed as mean ± standard deviation (SD). The effects of independent variables on dependent variables were explored with the help of the non-parametric percentile bootstrap method proposed by Hayes (2022) using the PROCESS (Version 4.1) macro model 6 [mediation effects test under 5,000 sampling conditions with 95% confidence intervals (CI), controlling for age and gender]. All analyses were conducted using IBM SPSS Statistics 26 software, with the significance level set at 0.05 and using two-sided tests (differences were considered statistically significant at *p* < 0.05).

## 4 Results

### 4.1 Common method bias test

Subjects conducted the distribution and collection of questionnaires through questionnaire star with a single method, there is a possibility of common method bias affecting the statistical results. Therefore, Harman one-way test (Howard et al., 2024) was conducted on the collected data. The results showed that there were five factors with eigenroots greater than 1. The first factor explained 36.99% of the variance, which was less than the critical value of 40%. Therefore, it is concluded that there is no serious problem of common method bias.

### 4.2 Demographic characteristics of the sample

As shown in Table 1, in the overall sample, 45.8% (542) were male students and 54.2% (642) were female students. Boys were significantly higher than girls in physical activity level and mental toughness, there was no significant difference in self-esteem between the two, and girls were significantly higher than boys in social support. As shown in Table 2, the overall sample had the largest proportion of university students aged 21 (32% of the total sample size, totaling 379 students) and the smallest proportion of university students aged 23 and older (3% of the total sample size, totaling 35 students). There was a significant difference in physical activity between ages, with 23-year-old university students being the highest and 19-year-old university students being the lowest. There was a significant difference in social support between ages, with 21-year-old university students being the highest and 20-year-old university students being the lowest.

**Table 1:** Gender differences.

Variable	Gender	Number (%)	*M*	*SD*	*t*	*p*
Physical activity	Male	542 (45.8)	31.76	23.51	10.35	0.00
	Female	642 (54.2)	19.03	18.79		
	Total	1,184 (100)	24.86	22.00		
Self-esteem	Male	542 (45.8)	29.66	5.67	0.08	0.93
	Female	642 (54.2)	29.64	4.74		
	Total	1,184 (100)	29.65	5.182		
Social support	Male	542 (45.8)	65.14	11.86	−2.70	0.01
	Female	642 (54.2)	66.98	11.57		
	Total	1,184 (100)	66.14	11.73		
Mental toughness	Male	542 (45.8)	28.17	6.94	5.14	0.00
	Female	642 (54.2)	26.19	6.36		
	Total	1,184 (100)	27.10	6.70		

**Table 2:** Age differences.

Variable	Age	Number (%)	*M*	*SD*	*F*	*p*
Physical activity	18	103 (8.7)	23.50	21.41	8.38	0.000
	19	325 (27.4)	22.10	20.26		
	20	268 (22.6)	25.62	21.28		
	21	379 (32)	23.78	21.96		
	22	74 (6.3)	33.15	25.62		
	23	35 (3.0)	42.83	25.50		
	total	1,184 (100)	24.86	22.00		
Self-esteem	18	103 (8.7)	29.36	5.90	1.98	0.08
	19	325 (27.4)	29.36	5.02		
	20	268 (22.6)	29.15	5.31		
	21	379 (32)	30.15	4.58		
	22	74 (6.3)	30.59	5.99		
	23	35 (3.0)	29.57	7.13		
	total	1,184 (100)	29.65	5.18		
Social support	18	103 (8.7)	68.61	15.24	8.40	0.00
	19	325 (27.4)	64.28	12.29		
	20	268 (22.6)	63.94	12.12		
	21	379 (32)	68.62	8.172		
	22	74 (6.3)	67.08	12.73		
	23	35 (3.0)	64.06	15.68		
	total	1,184 (100)	66.14	11.73		
Mental toughness	18	103 (8.7)	27.00	7.45	1.46	0.20
	19	325 (27.4)	26.35	6.73		
	20	268 (22.6)	27.16	6.34		
	21	379 (32)	27.43	6.54		
	22	74 (6.3)	28.09	6.88		
	23	35 (3.0)	28.00	7.97		
	total	1,184 (100)	27.10	6.70		

### 4.3 Descriptive statistics and correlation analysis of variables

As shown in Table 3, the correlation coefficients of physical activity, self-esteem, social support and psychological toughness are statistically significant. The correlation analysis shows that there is a positive correlation between physical activity and self-esteem, social support and psychological. The correlation between self-esteem and social support and mental toughness is noteworthy and it suggests to the present study that improving self-esteem and social support among university students may help improve their mental toughness. These findings provide initial support for the hypothesis of this study.

**Table 3:** Variable descriptive statistics and correlation analysis.

Variable	*M*	*SD*	1	2	3	4
Physical activity	24.86	22.00	1			
Self-esteem	29.65	5.18	0.16**	1		
Social support	66.14	11.73	0.10**	0.41**	1	
Mental toughness	27.10	6.70	0.22**	0.49**	0.44**	1

**p < 0.001.

### 4.4 Analysis of mediated effects

**Figure 2:**
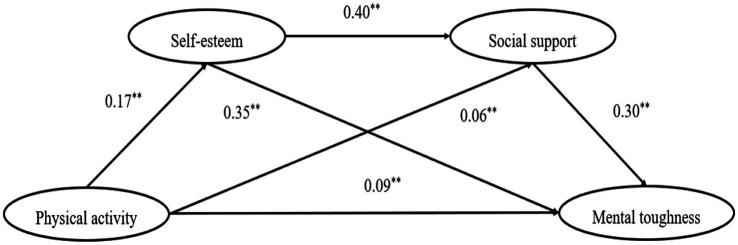
The chained mediating effect of self-esteem (M1) and social support (M2) in physical activity (X) and mental toughness (Y).

A mediated effects test was conducted according to the non-parametric percentile bootstrap method proposed by Hayes using the PROCESS (Version 4.1) macro model6 with a confidence interval (CI) of 95%, controlling for age and gender, under the 5,000-sampling condition (Hayes, 2022). The results of the data (see Table 4) showed that physical activity was positively associated with the direct path to mental toughness *β* = 0.09, *p* < 0.01, CI [0.04, 0.13], and Hypothesis 1 was tested. Second, the test of the mediating effect of self-esteem and social support between physical activity and mental toughness showed that physical activity was positively associated with self-esteem, *β* = 0.17, *p* < 0.01, CI [0.11, 0.23], and self-esteem was positively associated with mental toughness, *β* = 0.35, *p* < 0.01, CI [0.30, 0.40], and Hypothesis 2 was tested. Physical activity was positively correlated with social support, *β* = 0.06, *p* < 0.05, CI [0.08, 0.11]; and social support was positively correlated with mental toughness, *β* = 0.30, *p* < 0.01, CI [0.25, 0.35], Hypothesis 3 was tested. Self-esteem and social support were positively correlated, *β* = 0.40, *p* < 0.01, CI [0.34, 0.45], Hypothesis 4 was tested. The mediation effects test showed (see Table 5) a significant simple mediation effect for self-esteem and social support and a significant chained mediation effect for self-esteem and social support. The chain mediation model is shown in Figure 2.

**Table 4:** Chain-mediated regression analysis of physical activity and mental toughness.

Variables	Self-esteem		Social support		Mental toughness	
	*β*	*t*	*β*	*t*	*β*	*t*
Physical activity	0.17	5.77**	0.06	2.26*	0.09	3.39**
Self-esteem			0.40	14.79**	0.35	13.45**
Social support					0.30	11.69**
*R* ^2^	0.03		0.18		0.35	
*F*	12.80		64.31		124.67	

**P < 0.001.

**Table 5:** Chain mediation effect test of self-esteem and social support on physical activity and mental toughness.

Type of effect	Efficiency value	Boot SE	Bootstrap 95% CI		Efficacy as a percentage of
			Lower limit	Upper bound	
Total effect	0.19	0.30	0.13	0.24	100%
Direct effect	0.09	0.25	0.36	0.13	45.69%
Physical activity – self-esteem – mental toughness	0.06	0.02	0.03	0.09	32.80%
Physical activity-social support-mental toughness	0.02	0.01	0.001	0.04	10.22%
Physical activity – Self-esteem – Social support – Mental toughness	0.02	0.01	0.01	0.03	11.29%
Total indirect effect	0.10	0.020	0.06	0.14	54.31%

## 5 Discussion

### 5.1 Physical activity and mental toughness

In this study, a significant positive correlation was found between physical activity and mental toughness (*r* = 0.22), which is consistent with evidence from previous studies, and hypothesis 1 was verified by the inclusion of the mediating variable with a significant direct path between physical activity and mental toughness (*p* < 0.01). This result is consistent with the results of previous studies. First, scholars have noted that physical activity associated with mental toughness through both physiological and psychological mechanisms (Li et al., 2023; Herbert, 2022). At the physiological level, physical activity has been associated with the release of endorphins and the regulation of neurotransmitter activity, which may contribute to improvements in brain function and structure, potentially enhancing an individual’s sense of internal control and ability to cope with stress (Hossain et al., 2024). A longitudinal study found that students who engaged in moderate-intensity physical activity on a weekly basis had significantly higher levels of mental toughness than those who did not (Hannan et al., 2015). On a psychological level, physical activity can help individuals to improve their coping strategies, social interaction skills and emotional regulation, thus enhancing self-efficacy and stress coping (Husain et al., 2024). It can be seen that the same is true for students, the better the physical activity of university students, the higher their level of mental toughness, and the more positively they can cope with academic and life stress. Second, existing theoretical frameworks provide complementary perspectives on the relationship between physical activity and psychological resilience. Stress buffering theory posits that engagement in physical activity correlates with improved stress adaptation through the development of enhanced physiological and psychological resource reserves (Lines et al., 2020). Concurrently, self-efficacy theory delineates a bidirectional association between physical exercise and psychological constructs, wherein sustained participation in physical activities demonstrates co-variation with elevated self-efficacy levels, which subsequently exhibit positive covariation with mental toughness indicators (Yu and Mu, 2023). It can be seen that the results of this study further validate the positive effect of physical exercise on mental toughness. Therefore, it is recommended that university students enhance their mental toughness through regular exercise to better cope with stress in study and life.

### 5.2 Mediating effects of self-esteem

The present study found that self-esteem mediated the relationship between physical activity and mental toughness (indirect effect is 0.06), i.e., physical activity was positively related to self-esteem, and self-esteem was positively related to mental toughness, which was consistent with evidence from previous studies and verified Hypothesis 2. First, it was noted that self-esteem, as a positive evaluation of an individual’s self-worth and competence, positively associated with the individual’s mental toughness traits of self-acceptance, self-responsibility, and self-maintenance (Doğrusever et al., 2022; Chen et al., 2022). Individuals with high self-esteem are more able to maintain emotional stability in the face of stress and recover quickly through positive coping strategies, thus enhancing mental toughness. Conversely, mental toughness can help individuals cope positively with unfavorable situations and stress, which further enhances self-esteem levels (Basson, 2021; Kocatürk and Çiçek, 2023). For university students, high self-esteem is associated with better coping with academic and life stressors, and it is also positively linked to greater psychological well-being, potentially through its relationship with mental toughness (Basson, 2021). According to self-determination theory, physical activity may support the maintenance and enhancement of self-esteem by fulfilling intrinsic psychological needs such as autonomy, competence, and relatedness. Engagement in physical activity can also contribute to individuals’ sense of achievement and self-efficacy, which are positively correlated with self-esteem levels (Shang et al., 2021). A substantial body of research has demonstrated a strong positive association between physical activity and self-esteem. Furthermore, self-efficacy theory highlights that individuals with higher self-esteem tend to demonstrate greater emotional stability, a more positive self-concept, and more effective coping strategies in the face of adversity. These characteristics are often associated with higher levels of mental toughness. Empirical studies have reported that university students who regularly participate in physical activity tend to show higher levels of both self-esteem and mental toughness compared to their less active peers (Husain et al., 2024; Deng et al., 2023). Taken together, these findings suggest a robust pattern of positive associations among physical activity, self-esteem, and mental toughness.

### 5.3 Mediating effect of social support

The results of this study indicate that social support mediates the relationship between physical activity and mental toughness (indirect effect is 0.02), which is consistent with previous research evidence that physical activity positively predicts social support, and that social support and mental toughness are positively correlated; Hypothesis 3 was tested. Although some of the standardized effects observed in this study were small (e.g., *β* = 0.06) for the path from physical activity to social support, such findings may still hold practical significance. As physical activity is a widely accessible and modifiable behavior, even small improvements in psychological outcomes associated with increased physical activity may accumulate and have meaningful implications over time or at a broader population level (Abelson, 1985). Second, the Buffering Hypothesis theorizes that social support serves as a buffer when individuals face stress and adversity, mitigating the negative effects of stress on mental health (Wang and Bu, 2023; Rex, 2023). Previous research has suggested that social support may enhance individuals’ coping capacity by offering emotional reassurance and practical assistance, which is positively associated with greater mental toughness (Mayordomo et al., 2021). Psychologically resilient individuals also appear more capable of forming and maintaining positive social relationships, which, in turn, further reinforces their resilience and ability to manage life’s challenges effectively. For university students, adequate social support—whether from family, friends, teachers, or peers—has been linked to better coping with academic and life stressors, and higher levels of mental toughness (Permatasari et al., 2021). Moreover, evidence indicates that participation in sports, particularly team sports, can foster social skills and psychological well-being through interpersonal interactions and cooperation. These experiences are often associated with increased perceived social support (Van Luchene and Delens, 2021). For instance, university students involved in team sports are more likely to receive support from teammates and coaches, which not only contributes to improved athletic performance but is also positively associated with psychological well-being (Eather et al., 2023; Lin and Xiong, 2020). Physical activity, therefore, may contribute to both physical fitness and psychological well-being by facilitating the acquisition of social support. Furthermore, some scholars have emphasized the dual role of social support in both directly and indirectly enhancing mental toughness and coping ability by providing emotional and instrumental resources (Husain et al., 2024; Wang and Bu, 2023). In this context, social support emerges as a potential mechanism through which physical activity may be associated with improved mental toughness. The findings of the present study lend additional support to the proposed mediating role of social support in the relationship between physical activity and mental toughness, reinforcing the interconnected nature of these variables.

### 5.4 Chain-mediated effects of self-esteem and social support

The results of this study indicate a positive correlation between self-esteem and social support, aligning with findings from previous research and providing empirical support for Hypothesis 4. Individuals with high self-esteem tend to be more independent and confident, which may enable them to utilize available social support more effectively when facing stress, thereby mitigating the negative effects of stressful events. Such individuals are also more likely to maintain emotional stability and adopt positive coping strategies during challenging situations (Lee, 2020). Prior research has further suggested that an individual’s level of self-esteem may influence both their need for and receptivity to social support (Mahadevan et al., 2019). In addition, the “buffering hypothesis” posits that social support helps individuals manage stress by offering emotional reassurance and practical resources, which may, in turn, contribute to increased self-esteem (Zhang et al., 2024). One study found that higher levels of self-esteem among university students are associated with greater ability to cope with academic and life stress, partly due to increased access to social support. This relationship may be explained by the fact that individuals with high self-esteem often exhibit confidence and approachability in social interactions, enabling them to build supportive interpersonal relationships (Liu et al., 2021). Additionally, it has been reported that individuals who regularly participate in physical activity generally display higher self-esteem, which may facilitate the acquisition of social support and further contribute to improved mental toughness (Li et al., 2023). Physical activity not only improves physical health but also cultivates teamwork, resilience, and stress relief, fostering a sense of accomplishment and personal identity. These outcomes may enhance individuals’ self-confidence and openness in social contexts, making them more perceptive to support from others (Deng et al., 2022). Social support, in turn, provides both emotional comfort and instrumental assistance, which are positively associated with greater mental toughness and adaptive functioning in the face of adversity (Zhang and Jiang, 2025). Therefore, for university students, engaging in physical activity not only improves physical well-being but also appears to be positively related to increased self-esteem and social support, which together contribute to greater psychological resilience. These findings suggest that self-esteem and social support jointly serve as important chain mediators in the relationship between physical activity and mental toughness.

### 5.5 Practical significance

This study integrates physical exercise into the theoretical framework of university students’ mental toughness, offering a nuanced examination of the relational mechanisms between physical activity and psychological resilience. By doing so, it not only broadens the theoretical perspective within the field of mental toughness research but also provides valuable practical implications for designing interventions aimed at enhancing mental toughness among university students. The findings indicate that physical activity is a significant correlational factor associated with mental toughness. Given the unique developmental stage and life circumstances of university students, they often encounter a range of psychological challenges, including academic stress, employment anxiety, interpersonal difficulties, and identity crises. As an accessible and evidence-based intervention, physical exercise presents a promising avenue for supporting students’ psychological adaptation and resilience. This study provides empirical support for the positive association between physical activity and mental toughness, thereby offering a scientific rationale for promoting physical exercise as a component of university mental health initiatives. From a practical standpoint, enhancing physical activity among university students requires a multi-faceted approach. First, it is important to raise awareness about the benefits of physical activity, clarify its purpose, and cultivate intrinsic motivation to establish lifelong exercise habits. Second, institutions and broader society should work collaboratively to create a supportive environment by offering accessible sports facilities, adequate equipment, and a culture that values physical activity. Furthermore, organizing diverse group-based sports activities can help expand students’ social networks, enhance social support, and facilitate the development of self-esteem. These psychosocial benefits gained through physical activity participation may, in turn, contribute to greater psychological resilience. Such comprehensive efforts hold substantial practical significance. They not only assist university students in coping more effectively with academic and life stressors but also reduce the risk of mental health problems such as anxiety and depression, thereby promoting overall psychological well-being.

### 5.6 Research shortcomings and prospects

Although this study reveals the mechanism of physical activity’s influence on university students’ mental toughness by constructing a chain mediation model, which provides important theoretical and practical value for understanding the causes of mental toughness, there are still some limitations. First, due to the limitations of the questionnaire method and cross-sectional study design, this study was unable to infer the causal relationship between the variables, and a longitudinal study can be used in the future to examine the dynamic relationship between physical activity and mental toughness in stages. Second, although this study examined the mediating effects of self-esteem and social support, it did not examine potential moderating variables (e.g., gender, type of physical activity) that may influence the strength or direction of the observed relationships. Future research is encouraged to incorporate these moderators to provide a more comprehensive understanding of the mechanisms involved. Third, the sample for this study was drawn exclusively from university student populations in Guangdong Province, which may limit the generalizability of the findings. Although the sample size was relatively large, the geographic concentration and lack of detailed consideration of key demographic and socioeconomic factors may restrict the representativeness of the results. Given that psychological constructs such as mental toughness are influenced by a range of contextual variables—including regional cultural norms, age, gender, and socioeconomic background. Future research should consider expanding the sampling framework to include more diverse populations across different provinces and educational contexts. Fourth, the internal consistency of the physical activity scale used in this study was relatively low (Cronbach’s alpha = 0.62), which may raise concerns about potential measurement error. Although the scale has been widely used in previous research (e.g., Hou, 2024; Li et al., 2022), the modest reliability suggests that the observed relationships involving physical activity should be interpreted with caution. Future research is encouraged to adopt more comprehensive or domain-specific instruments with higher internal consistency to improve measurement accuracy and reduce potential bias.

## 6 Conclusion

(1) Physical activity is significantly and positively related to mental toughness; (2) physical activity positively predicts self-esteem and social support, and self-esteem and social support positively predict mental toughness. (3) Self-esteem and social support play a significant independent mediating effect between physical activity and mental toughness; (4) self-esteem and social support have a chain mediating effect between physical activity and mental toughness. These findings can provide empirical support for related scholars to study the relevant research in the area of physical activity and mental toughness among university students.

## Data Availability

The raw data supporting the conclusions of this article will be made available by the authors, without undue reservation.

## References

[ref1] Abelson R. P. (1985). A variance explanation paradox: when a little is a lot. Psychol. Bull. 97, 129–133. doi: 10.1037/0033-2909.97.1.129

[ref2] Auttama N. Seangpraw K. Ong-Artborirak P. Tonchoy P. (2021). Factors associated with self-esteem, resilience, mental health, and psychological self-care among university students in Northern Thailand. J. Multidiscip. Healthc. 14, 1213–1221. doi: 10.2147/JMDH.S308076, PMID: 34079280 PMC8166326

[ref3] Basson M. (2021). Coping, resilience, self-esteem and age as predictors of psychological well-being amongst undergraduate university students (unpublished doctorial dissertation). University of the free state, Bloemfontein.

[ref4] Campbell-Sills L. Stein M. B. (2007). Psychometric analysis and refinement of the Connor-Davidson Resilience Scale (CD-RISC): validation of a 10-item measure of resilience. J. Trauma. Stress. 20, 1019–1028. doi: 10.1002/jts.20271, PMID: 18157881

[ref5] Chen S. S. He Y. Xie G. D. Chen L. R. Zhang T. T. Yuan M. Y.. (2022). Relationships among adverse childhood experience patterns, psychological resilience, self-esteem and depressive symptoms in Chinese adolescents: a serial multiple mediation model. Prev. Med. 154:106902. doi: 10.1016/j.ypmed.2021.106902, PMID: 34863811

[ref6] Deci E. L. Ryan R. M. (2000). The “what” and “why” of goal pursuits: Human needs and the self-determination of behavior. Psychol. Inq. 11, 227–268. doi: 10.1207/S15327965PLI1104_01

[ref7] Deng J. Liu Y. Chen R. Wang Y. (2023). The relationship between physical activity and life satisfaction among university students in China: the mediating role of self-efficacy and resilience. Behav. Sci. 13:889. doi: 10.3390/bs13110889, PMID: 37998636 PMC10669265

[ref8] Deng C. Yu Q. Luo G. Lu S. (2022). Effects of 16 weeks of cheerleading on physical self-esteem and mental health of female college students. Front. Psychol. 13:925162. doi: 10.3389/fpsyg.2022.925162, PMID: 35800949 PMC9255666

[ref9] Doğrusever C. Türk N. Batmaz H. (2022). The mediating role of meaningful life in the relationship between self-esteem and psychological resilience. İnönü Üniversitesi Eğitim Fakültesi Dergisi 23, 910–928. doi: 10.17679/inuefd.1029866

[ref10] Eather N. Wade L. Pankowiak A. Eime R. (2023). The impact of sports participation on mental health and social outcomes in adults: a systematic review and the ‘Mental Health through Sport’ conceptual model. Syst. Rev. 12:102. doi: 10.1186/s13643-023-02264-8, PMID: 37344901 PMC10286465

[ref11] Gloria C. T. Steinhardt M. A. (2016). Relationships among positive emotions, coping, resilience and mental health. Stress Health 32, 145–156. doi: 10.1002/smi.2589, PMID: 24962138

[ref12] Hannan T. E. Moffitt R. L. Neumann D. L. Thomas P. R. (2015). Applying the theory of planned behavior to physical activity: The moderating role of mental toughness. J. Sport Exerc. Psychol. 37, 514–522. doi: 10.1123/jsep.2015-0074, PMID: 26524097

[ref13] Hayes A. F. (2022). Introduction to mediation, moderation, and conditional process analysis: a regression-based approach. 3rd Edn. New York, NY: The Guilford Press.

[ref14] Herbert C. (2022). Enhancing mental health, well-being and active lifestyles of university students by means of physical activity and exercise research programs. Front. Public Health 10:849093. doi: 10.3389/fpubh.2022.849093, PMID: 35548074 PMC9082407

[ref15] Hossain M. N. Lee J. Choi H. Kwak Y. S. Kim J. (2024). The impact of exercise on depression: how moving makes your brain and body feel better. Phys. Activity Nutr. 28, 43–51. doi: 10.20463/pan.2024.0015, PMID: 39097997 PMC11298280

[ref16] Hou X. M. (2024). Relationship between physical activity and self-esteem, self-efficacy, and depression in college students. Sports Sci. Technol. 45, 59–60+63.

[ref9001] Howard M. C. Boudreaux M. Oglesby M. (2024). Can Harman’s single-factor test reliably distinguish between research designs? Not in published management studies. Eur. J. Work Organ. Psychol 33, 790–804.

[ref17] Husain H. Samsudin S. Ayub A. F. M. Ahmad M. F. Afwan N. S. Z. S. (2024). A systematic literature review on the impact of participation in sport and physical activities on psychological resilience. Int. J. Public Health Sci. 13, 1727–1737. doi: 10.11591/ijphs.v13i4.24345

[ref18] Ji F. Y. Yu X. (1993). The self-esteem scale (SES). Chin. J. Ment. Health 7, 251–252.

[ref19] Jiang Y. Yi Z. Yao Y. Hu Y. Li F. Ma H. (2023). Effects of college students' mindfulness on depression symptoms during the epidemic prevention and control period: The mediating effect of psychological resilience. Front. Psych. 13:991449. doi: 10.3389/fpsyt.2022.991449, PMID: 36684002 PMC9845594

[ref20] Karunarathne R. A. I. C. (2022). Impact of perceived social support and social skills on adolescent’s self-esteem: the social support theory perspective. J. Bus. Technol. 6, 37–50. doi: 10.4038/jbt.v6i2.87

[ref21] Kim I. Ahn J. (2021). The effect of changes in physical self-concept through participation in exercise on changes in self-esteem and mental well-being. Int. J. Environ. Res. Public Health 18:5224. doi: 10.3390/ijerph18105224, PMID: 34069040 PMC8157161

[ref22] Kocatürk M. Çiçek İ. (2023). Relationship between positive childhood experiences and psychological resilience in university students: the mediating role of self-esteem. J. Psychol. Couns. Sch. 33, 78–89. doi: 10.1017/jgc.2021.16

[ref23] Lee K. (2020). Social support and self-esteem on the association between stressful life events and mental health outcomes among college students. Soc. Work Health Care 59, 387–407. doi: 10.1080/00981389.2020.1772443, PMID: 32538324

[ref24] Li Y. Guo K. (2023). Research on the relationship between physical activity, sleep quality, psychological resilience, and social adaptation among Chinese college students: A cross-sectional study. Front. Psychol. 14:1104897. doi: 10.3389/fpsyg.2023.1104897, PMID: 36844303 PMC9950505

[ref25] Li N. Wang D. Zhao X. Li Z. Zhang L. (2024). The association between physical exercise behavior and psychological resilience of teenagers: an examination of the chain mediating effect. Sci. Rep. 14:9372. doi: 10.1038/s41598-024-60038-1, PMID: 38654069 PMC11039466

[ref26] Li H. Y. Yan J. Shen B. Chen A. Huang C. (2022). Effects of extracurricular physical activity on life satisfaction of upper elementary school students: chain mediation of self-confidence and mental toughness. Chin. Sports Sci. Technol. 58, 51–56.

[ref27] Li N. Zhao S. Liu C. Dai K. Huang W. (2023). Exploring the relationship between perceived social support and college students’ autonomous fitness behavior: Chain mediating effect test. Front. Psychol. 13:1036383. doi: 10.3389/fpsyg.2022.1036383, PMID: 36817388 PMC9928751

[ref28] Liang D. C. (1994). Stress level of college students and its relationship with physical activity. Chin. J. Ment. Health 8, 5–6.

[ref29] Lin J. Y. Xiong H. (2020). A review of research on social support system for women's sports participation and its prospects. J. Chengdu Inst. Phys. Educ. 46, 21–26.

[ref30] Lines R. L. Ducker K. J. Ntoumanis N. Thøgersen-Ntoumani C. Fletcher D. McGarry S.. (2020). Stress, physical activity, and resilience resources: tests of direct and moderation effects in young adults. Sport Exerc. Perform. Psychol. 9:418. doi: 10.1037/spy0000152

[ref31] Liu Q. Jiang M. Li S. Yang Y. (2021). Social support, resilience, and self-esteem protect against common mental health problems in early adolescence: a non-recursive analysis from a two-year longitudinal study. Medicine 100:e24334. doi: 10.1097/MD.0000000000024334, PMID: 33530225 PMC7850671

[ref32] Mahadevan N. Gregg A. P. Sedikides C. (2019). Is self-regard a sociometer or a hierometer? Self-esteem tracks status and inclusion, narcissism tracks status. J. Pers. Soc. Psychol. 116, 444–466. doi: 10.1037/pspp0000189, PMID: 29608073

[ref33] Mayordomo T. Viguer P. Sales A. Satorres E. Meléndez J. C. (2021). “Resilience and coping as predictors of well-being in adults” in Mental health and psychopathology. ed. Rokach A. (London: Routledge), 265–277.

[ref34] Nimitniwat S. (2011). Development of non-formal education activities based on neo-humanist concept and collaborative learning to develop adversity quotient of students in private universities. Scholar 3, 49–54. Available at: https://assumptionjournal.au.edu/index.php/Scholar/article/view/258

[ref35] Ou Y. Y. Wang Y. H. Zhao H. M. (2023). The relationship between positive psychological qualities and psychological resilience in college students: the parallel mediating roles of social support and cell phone dependence. Adv. Psychol. 13, 6245–6263. doi: 10.12677/AP.2023.1312797

[ref36] Permatasari N. Ashari F. R. Ismail N. (2021). Contribution of perceived social support (peer, family, and teacher) to academic resilience during COVID-19. Golden Ratio Soc Sci Educ. 1:1. doi: 10.52970/grsse.v1i1.94

[ref37] Rex D. L. C. (2023). Perceived social support and sense of coherence as predictors of psychological well-being amongst university students during a pandemic (Unpublished master’s dissertation). University of the free state, Bloemfontein.

[ref38] Rodríguez-Romo G. Acebes-Sánchez J. García-Merino S. Garrido-Muñoz M. Blanco-García C. Diez-Vega I. (2022). Physical activity and mental health in undergraduate students. Int. J. Environ. Res. Public Health 20:195. doi: 10.3390/ijerph20010195, PMID: 36612516 PMC9819335

[ref39] Rosenberg M. (1965). Society and the adolescent self-image. Washington, D.C., U.S: Princeton University Press (APA).

[ref40] Shang Y. Xie H. D. Yang S. Y. (2021). The relationship between physical exercise and subjective well-being in college students: The mediating effect of body image and self-esteem. Front. Psychol. 12:658935. doi: 10.3389/fpsyg.2021.658935, PMID: 34122243 PMC8194825

[ref41] Southwick S. M. Sippel L. Krystal J. Charney D. Mayes L. Pietrzak R. (2016). Why are some individuals more resilient than others: the role of social support? World Psychiatry 15, 77–79. doi: 10.1002/wps.20282, PMID: 26833614 PMC4780285

[ref42] Stoltz P. (1997). Adversity quotient: turning obstacles into opportunities. USA: John Wiley & Sons, Inc.

[ref43] Stoltz P. (2000). Adversity quotient@work. USA: Harper Collins Publishers, Inc.

[ref44] Van Luchene P. Delens C. (2021). The influence of social support specific to physical activity on physical activity among college and university students: A systematic review. J. Phys. Act. Health 18, 737–747. doi: 10.1123/jpah.2020-0713, PMID: 33883289

[ref45] Wang S. M. Bu H. B. (2023). Effects of physical activity on adolescents' socioemotional competence-the chain-mediated role of social support and mental toughness. Res. Phys. Educ. 37, 24–33.

[ref46] Wei X. Lai Z. Tan Z. Ou Z. Feng X. Xu G.. (2024). The effect of physical activity on depression in university students: the mediating role of self-esteem and positive psychological capital. Front. Psychol. 15:1485641. doi: 10.3389/fpsyg.2024.1485641, PMID: 39380753 PMC11458536

[ref47] White R. L. Babic M. J. Parker P. D. Lubans D. R. Astell-Burt T. Lonsdale C. (2017). Domain-specific physical activity and mental health: a meta-analysis. Am. J. Prev. Med. 52, 653–666. doi: 10.1016/j.amepre.2016.12.008, PMID: 28153647

[ref48] Wu Y. Sang Z. Q. Zhang X. C. Margraf J. (2020). The relationship between resilience and mental health in Chinese college students: a longitudinal cross-lagged analysis. Front. Psychol. 11:108. doi: 10.3389/fpsyg.2020.00108, PMID: 32116918 PMC7012791

[ref49] Ye Y. M. Dai X. Y. (2008). Development of a social support scale for college students. Chin. J. Clin. Psychol. 16, 456–458.

[ref50] Yu H. Mu Q. (2023). Effect of physical exercise on negative emotions in adolescent: a chain-mediated effect of self-efficacy and mental toughness [Epubh ahead of preprint]. doi: 10.21203/rs.3.rs-3162324/v1

[ref51] Zeng X. H. Jie P. Wen Y. Luo L. Peng L. Fang P. (2023). The current situation and influencing factors of college students' mental toughness. Psychol. Monthly 18, 92–94.

[ref52] Zhang J. X. Jiang W. (2025). The relationship between social support and adolescents' problem behaviors: the mediating role of psychological resilience and the moderating role of parent-child attachment. J. Chengdu Normal College 32, 43–57.

[ref53] Zhang J. Lin X. Zhou S. Ye B. Zhang Y. Xiong H. (2024). Social support and parenting self-efficacy in parents of children with ASD: the mediating role of post-traumatic growth and the moderating role of self-esteem. Curr. Psychol. 43, 9025–9040. doi: 10.1007/s12144-023-05031-z

[ref54] Zhou H. Zhou Q. Y. (2022). Physical activity empowers college students' subjective well-being: the mediating role of cognitive reappraisal and mental toughness. J. Shandong Inst. Phys. Educ. 38, 105–111.

